# Roles of tick-cofeeding hedgehogs in the natural transmission of spotted fever group *Rickettsia*

**DOI:** 10.1371/journal.pntd.0013224

**Published:** 2025-09-15

**Authors:** Haiming Yi, Weilin Huang, Changqiang Zhu, Yixin Ge, Xijing Yang, Sunjie Yang, Chuchu Ye, Junhu Wang, Qiong Chen, Yingqing Mao, Hongming Wang, Lele Ai, Wei Guo, Chao Chen, Weilong Tan, Yuexi Li, Yong Qi

**Affiliations:** 1 Huadong Research Institute for Medicine and Biotechniques, Nanjing, Jiangsu, China; 2 Institute of Rocket Force Medicine, State Key Laboratory of Trauma, Burns and Combined Injury, Third Military Medical University (Army Medical University), Chongqing, China; 3 School of Engineering, China Pharmaceutical University, Nanjing, Jiangsu, China; 4 School of Life Science, Xuzhou Medical University, Xuzhou, Jiangsu, China; Egerton University, KENYA

## Abstract

Tick-borne spotted fever group *Rickettsia* (SFGR) poses a significant threat to public health worldwide. The cofeeding behavior of ticks attaching host animals, such as hedgehogs, has been identified as a potential mechanism for transmitting SFGR between infected and uninfected ticks, potentially increasing the prevalence of SFGR. However, the overall positive rate of SFGR in free-living ticks remains relatively low, suggesting that the role of tick-cofeeding in amplifying SFGR prevalence may not be as substantial as previously believed. To explore the impact of tick-cofeeding hedgehogs on the natural transmission of SFGR, to clarify the underlying hypotheses, and to provide robust data to support targeted prevention and control strategies for spotted fever, this study developed a transmission model using tick-cofeeding hedgehogs that simulates the natural transmission process. Both *Rickettsia*-infected and uninfected tick populations were established and used for cofeeding on mice or hedgehogs. Among formerly uninfected nymphs that cofed on mice, 75.61% acquired *Rickettsia* after engorgement, but this infection rate dropped sharply to 9.68% after molting. In contrast, formerly uninfected adults that cofed on hedgehogs showed a 100% infection rate after engorgement. However, the infection rates declined significantly in their offspring, with only 11.12% of normal-hatching eggs and 3.12% of larvae testing positive. Additionally, we observed mortality in infected engorged adults and their eggs. Our results demonstrate that while tick-cofeeding on hedgehogs can lead to a high positive rate of *Rickettsia* in ticks, the infections acquired through cofeeding fail to sustain this high positivity rate due to several mechanisms. Firstly, rickettsiae obtained through cofeeding or blood meals do not consistently establish infections in all recipient ticks, resulting in a significant decline in positive rates as ticks progress to subsequent developmental stages. Secondly, adult ticks infected via cofeeding tend to reduce the infection rate in their offspring through various mechanisms, including tick mortality caused by rickettsiae, egg hatching failure, and a low transovarial transmission rate. Additionally, in natural settings, infections from other pathogens may similarly contribute to tick mortality and reduced egg hatching. This study elucidates why rickettsiae maintain a low prevalence in nature and evaluates the actual effects of tick-cofeeding on pathogen distribution among ticks. While tick-cofeeding on host animals have been considered important amplifiers of SFGR prevalence, our findings indicate that their impact is not as significant as previously assumed.

## Author summary

Ticks can carry and transmit various pathogens, including spotted fever group *Rickettsia* (SFGR). SFGR comprises at least 20 pathogens that can cause spotted fever (SF) in humans, posing significant public health threats. As a naturally occurring zoonotic pathogen, SFGR primarily affects small mammals. It is generally believed that under natural conditions, SFGR can spread from one tick to dozens of ticks through the infection pathway of “infected tick-host-uninfected tick”. Theoretically, this process could not only sustain the presence of SFGR in nature but also effectively increase the prevalence of the bacteria in ticks. However, in reality, the positive rate of SFGR in free-living ticks in nature has not become extremely high, contradicting the hypothesis. We speculate that the role of tick-cofeeding on small mammals in amplifying SFGR prevalence may not be as significant as previously assumed and that unknown mechanisms may contribute to the observed low positivity rates in ticks in nature. In this study, we established a tick-animal transmission model to simulate this natural process and analyzed why the positive rate in ticks remains at a relatively stable level.

## Introduction

Spotted fever group *Rickettsia* (SFGR) comprises a large array of rickettsial species—at least 47—over 20 of which are known to cause spotted fever (SF) in humans [1]. The ongoing identification of new SFGR species continues to pose significant public health threats. In Southeast Asia, for example, rickettsial disease ranks as the second most common cause of non-malaria related fever, following dengue fever [2,3].

As a naturally occurring zoonotic pathogen, SFGR primarily affects small mammals, including rodents, insectivores, lagomorphs, and some bird species [1,4]. In China, key reservoir hosts include the Eastern field mouse, black-striped grass mouse, house mouse, and goats [2]. Additionally, a variety of wild animals, such as bats, coyotes, stone martens, raccoons, chimpanzees, macaques, wild boars, antelopes, and hedgehogs, as well as domestic livestock including cattle, cats, dogs, horses, camels, and yaks, are known to carry rickettsiae [1], although the roles these animals play in the transmission of SFGR are not well understood.

Typically, a host animal is infested by a few to dozens of ticks, which may be infected or uninfected. Cofeeding transmission facilitates the transfer of SFGR between infected and uninfected ticks [5]. For instance, Lee et al. demonstrated that *Rickettsia parkeri* transmission between infected and uninfected *Amblyomma maculatum* ticks was more efficient when ticks cofed in close proximity on beef calves [6]. Another laboratory study confirmed the transmission of *R. parkeri* between *Amblyomma americanum* and *A. maculatum* ticks when cofeeding on guinea pigs [7].

In our earlier investigation, we assessed the molecular prevalence of SFGR in ticks collected from wild hedgehogs and domestic bovines. Notably, the positive rate for SFGR in ticks from hedgehogs (83.2%) was significantly higher than in ticks from bovine (1.2%) [8]. This suggests that animals like hedgehogs may serve as a “bridge” in transmitting SFGR from infected ticks to uninfected ones during cofeeding. The efficiency of SFGR transmission via tick cofeeding may vary based on the specific pathogen, tick vector, and host [6]. Our prior study indicated that the transmission efficiency of SFGR in ticks cofeeding on wild hedgehogs is quite high. Theoretically, this process could not only sustain the presence of SFGR in nature but also effectively increase the prevalence of the bacteria in ticks.

However, despite the rising density of hedgehogs in urbanizing areas of China [9], the actual prevalence of SFGR in free-living ticks remains relatively low, contradicting our hypothesis [10]. We speculate that the role of tick-cofeeding on hedgehogs in amplifying SFGR prevalence may not be as significant as previously assumed and that unknown mechanisms may contribute to the observed low positivity rates in ticks in nature.

*Haemaphysalis flava* is a tick species widely distributed in the East and South Asia. Like other *Haemaphysalis* spp., *H. flava* is a three-host tick with a life cycle including four stages: egg, larva, nymph, and adult. The host species of *H. flava* are extensive, and it has been typically found on the body surface of hedgehogs [11–13]. In recent years, *H. flava* has drawn attention as a potential competent vector of various pathogens, like severe fever with thrombocytopenia syndrome virus, SFGR, *Anaplasma*, *Coxiella*, etc. [13,14]. Several SFGR species have been detected in *H. flava*, including *Rickettsia heilongjiangensis*, *Rickettsia japonica*, and *Rickettsia raoultii* [8,13], indicating *H. flava* is suitable for exploring the role of tick-cofeeding on hedgehogs in amplifying SFGR prevalence*.*

*R. heilongjiangensis*, the causative agent of Far-Eastern spotted fever (FESF) for humans, has been found to be prevalent mainly in North and East Asia, including China, Siberia and far-eastern Russia, and Japan [15–18]. The patient’s symptoms are relatively mild, including fever, chill, headache, dizziness, myalgia, arthralgia, and anorexia [16], while *R. heilongjiangensis* caused severe systemic infection in mouse models [19]. In our previous study, the main SFGR species identified in hedgehog-attached ticks was *R. heilongjiangensis* [8].

**Fig 1:**
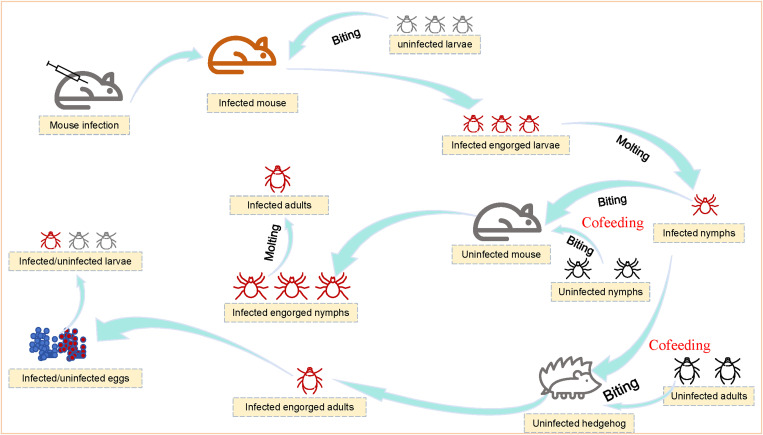
The designed *Ricksttsia*-tick-hedgehog transmission model. (1) To obtain infected nymphs, firstly, *Rickettsia heilongjiangensis* was used to infect mice, and uninfected larvae were infected through biting the infected mice. Then the *R. heilongjiangensis*-infected engorged larvae molted to *R. heilongjiangensis*-infected nymphs. (2) The infected nymphs and uninfected nymphs cofed uninfected mice, and the horizontal transmission efficiency was evaluated between the infected nymphs and uninfected nymphs. Also, the transstadial transmission efficiency was evaluated from the infected engorged nymphs to adults. (3) The infected nymphs and uninfected adults cofed uninfected hedgehogs, and the horizontal transmission efficiency was evaluated between the infected nymphs and uninfected adults, and the transovarial transmission efficiency was evaluated from infected adults to their eggs as well as the offspring larvae.

To investigate the roles of tick-cofeeding on hedgehogs in the natural transmission of SFGR, the present study has developed a *Rickettsia*-tick-hedgehog model using *R. heilongjiangensis*, *H. flava* ticks, and hedgehogs (Fig 1). This model aims to elucidate the underlying mechanisms and provide comprehensive data to support targeted prevention and control strategies for spotted fever.

## Materials and methods

### Ethic statement

The use of animal samples in this study was reviewed and approved by the Ethics Committee of the Huadong Research Institute for Medicine and Biotechniques. All animal care and treatments adhered to the committee’s standards, and every effort was made to minimize suffering.

### Rickettsia culture

*Rickettsia heilongjiangensis* was utilized as a representative of SFGR in this study. Vero cells were cultured in RPMI-1640 media supplemented with 10% fetal bovine serum (FBS) in a 37°C, 5% CO_2_ incubator. Once the cells reached approximately 80% confluence, the culture media was changed to one containing 3% FBS. *R. heilongjiangensis*, stored in liquid nitrogen, was thawed and introduced into the culture medium for infection. The infected cells were incubated at 33°C until a cytopathic effect affecting over 50% of the cells was observed. The cells were then scraped and stored at -80°C. The concentration of *R. heilongjiangensis* was determined using real-time fluorescent quantitative PCR (qPCR) as previously described (Table 1) [19].

**Table 1:** Primers and probes used in the present study.

Methods	Targets	Primers and Probes
Real-time fluorescent quantitative PCR (qPCR)	*ompB* gene of *Rickettsia heilongjiangensis*	Forward Primer: ATCTGAAGCGGGAGCAATACC; Reverse Primer: CATCAGTATAGAAAGGTTGCCCATA; Probe: FAM-TCATTATCAACAGCCTCGTCA-P.
Semi-nested pcr PCR	17-kDa outer membrane protein gene	Forward Primer 1: TTTACAAAATTCTAAAAACCAT; Forward Primer 2: GCTCTTGCAACTTCTATGTT; Reverse Primer: TCAATTCACAACTTGCCATT.
PCR	Cytochrome c oxidase subunit 1 (*cox1*) gene of ticks	Forward Primer: GAGTATCAATGTCTATCCCTACTGTAAATATGTGATGT; Reverse Primer: AACAATATATTTAATTTTTGGTGCTTGAGCCG.

### Establishment of an uninfected population of ticks

Wild hedgehogs were captured with the help of local villagers in Xuyi County, Jiangsu Province. Ten engorged adult ticks of *H. flava* were collected from their body surface. Each tick was placed in a 50 mL centrifuge tube with perforated caps and incubated in an artificial climate chamber at 28°C with 85% relative humidity until they laid eggs. Larvae hatched from the eggs approximately 2–3 weeks later.

Each adult tick (after laying eggs) and its offspring larvae (approximately 30 larvae per parent tick) were rinsed twice in 75% alcohol and once in sterilized phosphate-buffered saline (PBS) (8.1 mM Na_2_HPO_4_, 1.9 mM NaH_2_PO_4_, 154 mM NaCl; pH 7.4). These larvae from the same tick were pooled. Each adult and the larvae pool were then separately homogenized in 1 mL of RPMI-1640 medium using a tissue homogenizer. Genomic DNA was extracted from 300 μL of each sample using a MagaBio Plus DNA Extraction Kit (Bioer Technology, Hangzhou, China). The extracted DNA was used as templates to detect the presence of *R. heilongjiangensis* using qPCR, as well as a semi-nested PCR assay targeting the 17-kDa outer membrane protein gene of SFGR (Table 1) [13,19]. The tick species was confirmed using PCR method targeting the cytochrome c oxidase subunit 1 (*cox1*) gene (Table 1), as described previously [12,20]. The PCR products were sequenced using the Sanger sequencing method and aligned with existing sequences deposited in Genbank database.

The larvae were kept to establish an uninfected tick population if both the parent tick and its larvae tested negative for *R. heilongjiangensis* and other SFGR species. After another 2–3 weeks of incubation, the remaining larvae were allowed to bite a mouse to become engorged. The subsequent nymphs were obtained after molting. After another 2–3 weeks, the nymphs were used to bite a mouse or rabbit to become engorged nymphs. Adults were then obtained following molting and used to bite a rabbit for engorged adults. The offspring eggs were collected after the adults laid eggs. Through this subculture process, *Rickettsia*-negative populations of each developmental stage were obtained for further experiments.

### Mouse infection model construction

Previous studies have reported *R. heilongjiangensis* infection models in both C3H/HeN and BALB/c mice [19,21]. To determine the more suitable strain for our study, we evaluated the potential of these two mouse strains to transmit *Rickettsia* to ticks via biting. Specific Pathogen Free (SPF) mice were purchased from Nanjing Kaisijia Biotechnology Company (Nanjing, China). For each mouse species, 21 female mice aged 4–6 weeks were selected, with 3 designated as the control group and the remaining 18 as the experimental group. Mice in the experimental group were intraperitoneally inoculated with the prepared *R. heilongjiangensis* cell solution at a dose of 300 μL per mouse, containing a copy number of 3.65 × 10^5^/μL (4.00 × 10^3^ plaque forming units/μL) according to preliminary experimental results. The control group received the same volume of sterile PBS.

Physiological status of the mice was observed and recorded daily. Mice in the experimental group were euthanized in groups of three on days 1–6 post-infection, while the control mice were euthanized on day 6. Serum (200 μL/mouse) and various organs (20 mg/mouse) including the heart, liver, spleen, lungs, kidneys, and brain were collected from each mouse for DNA extraction as described above. The concentration of *R. heilongjiangensis* in each sample was tested using qPCR as previously described.

### Establishment of an infected population of nymphs

To generate a population of infected ticks, selected C3H/HeN mice were infected with *R. heilongjiangensis* as described above, and about 150 questing larvae were immediately allowed to bite the three infected mice, 50 ticks per mouse. After the larvae engorged and detaches themselves, genomic DNA from 10 ticks was extracted separately to detect *R. heilongjiangensis* using semi-nested PCR as mentioned above. The remaining engorged larvae were incubated until molting into nymphs. *R. heilongjiangensis* was similarly detected in 10 nymphs to assess the infection rate.

### Infection of mouse through tick bite

Nine C3H/HeN mice were infested with ticks from the infected nymph population, ensuring that at least 5 ticks attached to each mouse. An additional three mice were bitten by five uninfected nymphs per mouse as control. The physiological status of the mice was monitored and recorded daily. Mice in the experimental group were euthanized in groups of three on days 1, 6, and 12 post-attachment, while the control group was euthanized on day 12. From each mouse, skin samples were collected at the site of tick bites, along with sera and various organs, including the heart, liver, spleen, lungs, kidneys, and brain. These samples were processed for DNA extraction, followed by detection of *R. heilongjiangensis* using semi-nested PCR as described above.

### Evaluation of the horizontal and transstadial transmission efficiency through tick-cofeeding in mice

To assess the potential role of mice in the transmission of *R. heilongjiangensis*, each C3H/HeN mouse was infested with at least five ticks from an infected nymph population. The infected nymph population served as donors for pathogen transmission, while uninfected ticks served as recipients. After the infected nymphs attached, their bite sites were marked, and approximately 10 uninfected nymphs per mouse were immediately introduced to feed on the same host. To distinguish between the two tick groups, the infected nymphs were collected just before natural detachment, while the uninfected nymphs were allowed to detach naturally before collection. Partial engorged nymphs (previous uninfected ones) were randomly collected for detection of *R. heilongjiangensis* via semi-nested PCR. The remaining engorged nymphs were incubated to allow for molting into adults. After two weeks of incubation, each adult tick was used to extract its genomic DNA, and the presence of *R. heilongjiangensis* was tested using semi-nested PCR to determine the infection rate.

### Evaluation of the horizontal and transovarial transmission efficiency through tick-cofeeding in hedgehogs

To evaluate the potential role of hedgehogs in the transmission of *R. heilongjiangensis*, pet hedgehogs (*Atelerix albiventris*) were employed. Female hedgehogs, approximately two months of age, were purchased from a local pet store. To confirm that the hedgehogs were free from SFGR, 200 μL of blood was drawn through their hearts into anticoagulant tubes using a syringe and subjected to DNA extraction for detection of SFGR using semi-nested PCR targeting the 17-kDa outer membrane protein gene as mentioned above. Meanwhile, 1 mL of blood was drawn from each hedgehog for serum separation. The blood was left at 4 °C overnight and centrifuged at 3 000 rpm for 10 min to obtain the serum.

Hedgehogs confirmed to be *R. heilongjiangensis*-negative were then conducted to test the presence of specific antibodies against *R. heilongjiangensis* in the sera using an indirect immunofluorescence assay (IFA)*.* Briefly, *R. heilongjiangensis* cultured with vero cells was coated onto slides, air-dried and fixed with cold acetone [22]. The bacteria on the slides were incubated with 100 μL of serially diluted hedgehog sera (1:10–1:80) in a moist chamber at 37°C for 30 min. After three rinses using PBS, the bacteria on the slides were incubated with fluorescein isothiocyanate (FITC)-conjugated protein A (0.5 μg/mL; XianRuixi Biotech Company, China) at 37°C for 30 min. After another three rinses, the slides were observed using a fluorescence microscope. A positive control, which used diluted serum (1:80 dilution) collected from a *R. heilongjiangensis*-immunized hedgehog was conducted. The hedgehog was previously immunized by intraperitoneal injection of inactivated *R. heilongjiangensis* twice, with two weeks interval. The positive serum was collected two weeks after the second immunization. The serum was considered negative for specific antibodies against *R. heilongjiangensis* when the bacteria on the slides were unstained with fluorescence at any dilutions.

Three hedgehogs tested negative for both *R. heilongjiangensis* DNA and specific antibodies were utilized for further experiments. Two hedgehogs were infested with a minimum of five ticks from the infected nymph population (donors) and five uninfected adult ticks (recipients) simultaneously. As a control, five uninfected nymphs and five uninfected adults were used for cofeeding on the other hedgehog. Male adult ticks were also simultaneously placed on the body surface of the hedgehogs, and the ratio of male to female was 1: 1. Following the bites, the engorged adult ticks were collected and incubated until they laid eggs. Each adult tick produced five groups of eggs (25 eggs per group) for further analysis, while the remaining eggs were incubated until they hatched into larvae. Samples from the adults (1 tick per group), eggs (25 per group), and larvae (20 per group) were homogenized for DNA extraction and subsequent detection of *R. heilongjiangensis* via semi-nested PCR. Additionally, donor nymphs were tested post-cofeeding to ensure that at least one tick in each group tested positive for *R. heilongjiangensis*.

### Evaluation of the transovarial transmission efficiency of SFGR in ticks from wild hedgehogs

To examine the transmission potential of wild hedgehogs, seven engorged adult ticks were collected from wild hedgehogs captured in Xuyi County, Jiangsu Province, in August 2024. The eggs from these ticks were incubated to hatch into offspring larvae. Detection of *R. heilongjiangensis* in the adult ticks, their eggs, and the offspring larvae was conducted using the methods described above.

### Statistical analysis

Bacterial loads in the organs of different mouse species were compared using two-way ANOVA with SAS 9.4 software, considering a *P* value of <0.05 as statistically significant. The positive rate of *Rickettsia* in tick pools was calculated utilizing maximum likelihood estimation (MLE) with the Excel add-in PooledInfRate version 4.0 statistical software package [23].

## Results

Here, we successfully established a sustainable breeding population of ticks free of SFGR. BLAST analysis of the tick *cox-1* gene sequence showed 100% identity with the *H. flava* reference sequence (MZ434945.1) in GenBank, confirming the species identification.

To establish a *Rickettsia* infection model via tick bite, we infected BALB/c and C3H/HeN mice using *R. heilongjiangensis* through intraperitoneal injection and compared the physiological status and changes in *Rickettsia* load in the sera and organs of both mouse strains. Both infected groups exhibited decreased activity levels, as well as signs of unease such as shrugging and arching, particularly evident between days 1 and 2 post-infection. These symptoms intensified from days 3–5, with C3H/HeN mice demonstrating more severe responses than BALB/c mice. No such physiological changes were observed in the control (uninfected) mice.

**Fig 2:**
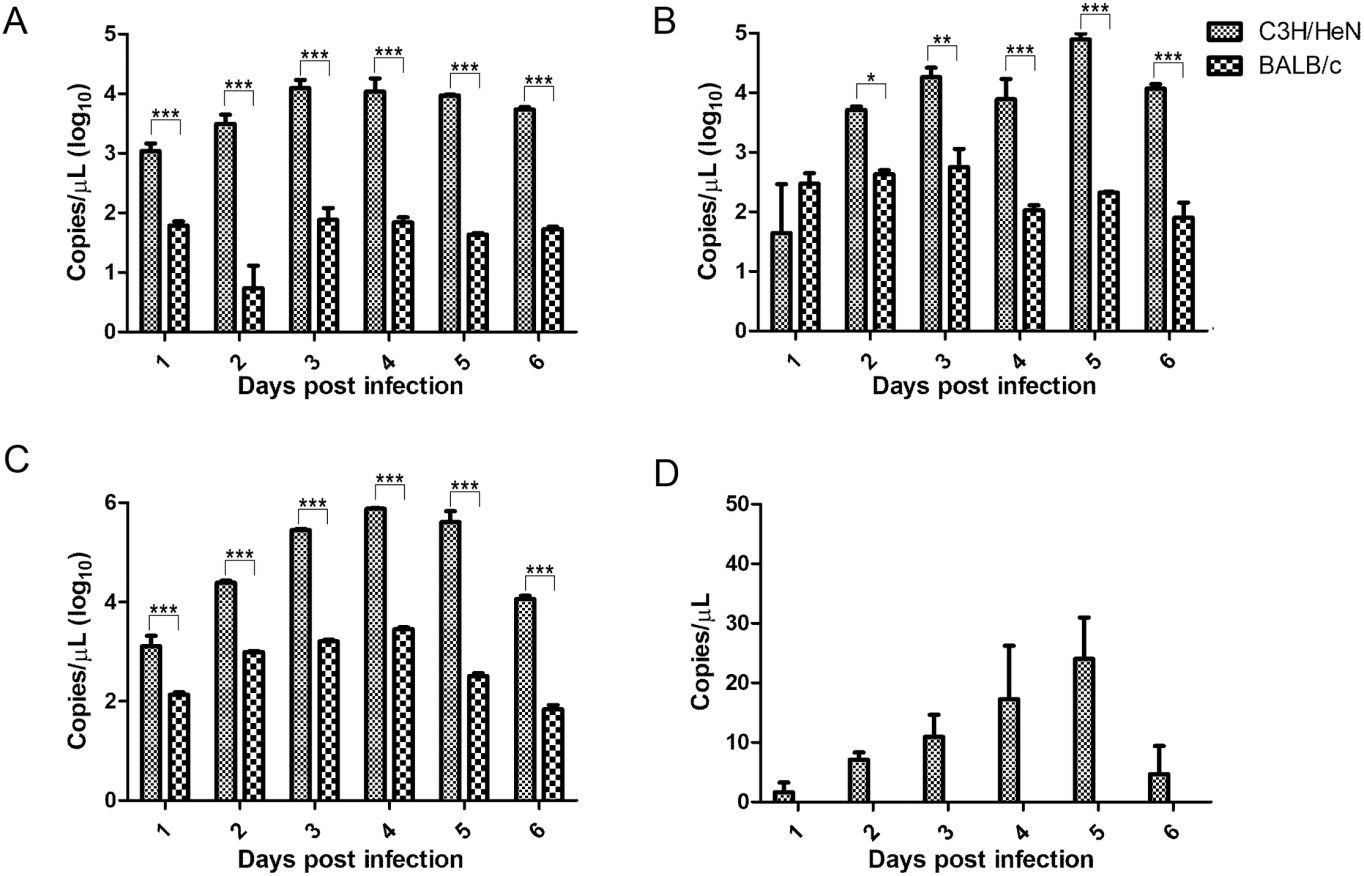
Loads of *Rickettsia heilongjiangensis* in different organs or sera of C3H/HeN and BALB/c mice post-infection. A, lungs; B, livers; C, spleens; D, sera. *, *P* < 0.05; **, *P* < 0.01; ***, *P* < 0.001. Differences between groups are considered significant when *P* < 0.05, as analyzed using two-way ANOVA.

The bacterial loads in the sera and organs of the infected mice were measured using qPCR. As illustrated in Fig 2, the bacterial loads in the sera and organs of C3H/HeN mice were significantly higher (*P* < 0.05) than those in BALB/c mice. Remarkably, *R. heilongjiangensis* was undetectable in the sera of infected BALB/c mice in this study. The bacterial loads peaked in the sera and organs of C3H/HeN mice between days 3 and 5 post-infection, leading to the conclusion that C3H/HeN mice were the preferred model for further studies.

To generate a population of *R. heilongjiangensis*-infected ticks, uninfected larvae were allowed to feed on infected C3H/HeN mice. Larval ticks typically feed for 4–5 days before they detach, with the highest bloodsucking efficiency observed during the last 1–2 days. Given that the peak bacterial loads in the sera of infected mice occur between days 3–5 (Fig 2D), the larvae were allowed to attach to the mice on the day of *Rickettsia* injection. The infection rates of the engorged larvae and their developing nymphs were found to be 100% (10/10) and 60% (6/10), respectively (Table 2), indicating successful development of a population of *R. heilongjiangensis*-infected nymphs.

**Table 2:** Positive rates of *R. heilongjiangensis* in various groups of ticks at different developmental stages used in the present study.

No.	Developmental stages	Pool size	Total pools or individuals	Positive pools or individuals	Positive rates^*^	Notes
1	Engorged larvae	1	10 individuals	10 individuals	100%	Feeding infected mice
2	Nymphs	1	10 individuals	6 individuals	60%	Developed from infected larvae of No. 1
3	Engorged nymphs	1	41 individuals	31 individuals	75.60%	Cofeeding mice simultaneously with nymphs of No. 2
4	Adults	1	31 individuals	3 individuals	9.68%	Developed from the remaining engorged nymphs of No. 3
5	Engorged adults	1	5 individuals	5 individuals	100%	Cofeeding hedgehogs with nymphs of No. 2; 1 adult died and 2 adults laid dead eggs.
6	Eggs	25	20 pools	20 pools	100%	Dead eggs laid by 2 adults in No. 5
7	Eggs	25	20 pools	5 pools	11.12% (4.19%-24.86%)^*^	Normal eggs laid by 2 adults in No. 5
8	Larvae	20	16 pools	1 pools	3.12% (0.18%-15.26%)^*^	Hatched from eggs of No. 7
9	Engorged adults	1	6 individuals	5 individuals	83.33%	Collected from wild hedgehogs; 2 positive adults died and 1 positive adult laid dead eggs.
10	Eggs	25	5 pools	1 pool	7.93% (0.48%-40.77%)^*^	Dead eggs laid by the adult in No. 9
11	Eggs	25	10 pools	9 pools	72.23% (36.72%-100%)^*^	Normal eggs laid by the 2 positive adults in No. 9
12	Larvae	20	16 pools	8 pools	32.63% (15.55%-63.54%)^*^	Hatched from eggs of No. 11; eight groups of positive larvae were from the same parent tick.

*Positive rates in pools were calculated by PooledInfRate and the 95% confidence interval was indicated in parentheses.

To evaluate the infection capability of this established population, nymphs were used to feed on C3H/HeN mice, ensuring that at least five nymphs fed on each mouse. With a 60% *R. heilongjiangensis* infection rate, using five nymphs provided a 99% probability that at least one tick would be positive. Nymphs typically attach for 5–7 days. After 1–3 days of feeding, mice in the experimental groups (infested with infected nymphs) exhibited similar symptoms to those in the control group (infested with uninfected nymphs). However, the experimental groups displayed decreased activity and signs of distress from day 4 onward, with conditions worsening between days 6 and 8 post-attachment, followed by gradual improvement. Using semi-nested PCR, *R. heilongjiangensis* was detected in the sera, skin (bite site), and spleens of mice at days 1, 6, and 12 post-attachment with infected nymphs, while the bacteria were undetectable in the livers, lungs, and kidneys (S1 Fig). All the mice in the experimental groups were positive for *R. heilongjiangensis.* These results indicate that the established population of infected ticks could successfully transmit *R. heilongjiangensis* to mice, resulting in bacteremia, with the spleen identified as a potential target organ.

To assess the horizontal transmission capability of mice as a “bridge” for transmitting *R. heilongjiangensis* from infected to uninfected ticks, uninfected nymphs were allowed to feed on mice previously attached by infected nymphs. As summarized in Table 2, 31 out of 41 engorged nymphs tested positive for *R. heilongjiangensis*, resulting in a positive rate of 75.61%. The remaining 31 engorged nymphs were allowed to molt. After molting, only 3 of the 31 developing adults remained positive, yielding a positive rate of 9.68%. Consequently, the transstadial transmission from nymphs to adults occurred, while the transmission efficiency was not high considering the sharply drop of positive rates.

Additionally, we evaluated the transmission efficiency of *R. heilongjiangensis* through cofeeding using hedgehogs. Uninfected adult ticks were employed, considering the noted phenomenon that most attached ticks found on hedgehogs are adults. Two hedgehogs were infested with both infected nymphs and uninfected adults. Five engorged adults were collected, resulting in four laying eggs while one died. The infection rate of these adult ticks was 100% (5/5, Tables 2 and 3). Notably, eggs laid by two adults did not hatch after two months of incubation, while the remaining two adults’ eggs hatched normally. The positive rates were assessed for the collected eggs: the unhatched egg group showed a 100% (20/20) positive rate, whereas 5 out of the 20 pools in the normal-hatching egg group were positive for *R. heilongjiangensis* (Tables 2 and 3), and the infection rate for the normal-hatching egg groups was estimated at 11.12% (95% confidence interval (CI): 4.19%-24.86%) calculated using the PooledInfRate tool. After hatching, 1 out of the 16 pools of larvae were positive for *R. heilongjiangensis*, with a positive rate of 3.12% (95% CI: 0.18%-15.26%) (Table 2). This indicates transovarial transmission of *Rickettsia* exists in *H. flava*, while the positive rate dramatically decreased from the eggs to the offspring larvae.

**Table 3:** The SFGR test results, along with the growth, development, and reproductive status of the engorged adult ticks and their offspring.

	Ticks cultured in the laboratory					Ticks from wild hedgehogs					
Tick No.	1	2	3	4	5	1	2	3	4	5	6
Positive for SFGR	P	P	P	P	P	P	N	P	P	P	P
Survival conditions	S	S	S	S	D	S	S	S	S	D	D
Offspring eggs (positive pools/total pools)	2/10	3/10	10/10	10/10	–	4/5	0/5	5/5	1/5	–	–
Offspring egg conditions	S	S	D	D	–	S	S	S	D	–	–
Offspring larvae (positive pools/total pools)	0/8	1/8	–	–	–	0/8	0/8	8/8	–	–	–

P, positive; N, negative; S, survival; D, dead

In another experiment, ten uninfected adult ticks were allowed to feed on uninfected hedgehogs. In stark contrast to the infected ticks, all these engorged uninfected adults survived and laid eggs normally, and all eggs hatched into larvae, demonstrating that *Rickettsia* infection adversely affected the survival, development, and reproductive success of engorged adult ticks.

To ascertain the actual transmission role of hedgehogs in the wild, we collected six engorged adult ticks from wild hedgehogs. Out of these, four ticks laid eggs while the other two died without reproducing (Table 3). Subsequent analysis using semi-nested PCR targeting the 17-kDa outer membrane protein gene revealed that both deceased ticks were positive for *R. heilongjiangensis*. Among the four ticks that laid eggs, three were *R. heilongjiangensis*-positive, while one tick and its eggs tested negative. One of the positive adults had eggs that failed to hatch, and only one out of five of its pooled eggs tested positive for *R. heilongjiangensis*, yielding a positive rate of 7.93% (95% CI: 0.48%-40.77%) as calculated using the PooledInfRate tool (Tables 2 and 3). Other factors, such as potential infections with different pathogens, may have affected the hatching ability of these eggs. Conversely, the eggs from the two remaining ticks hatched successfully, with 9 out of 10 egg pools testing positive (Table 3), resulting in a positive rate of 72.23% (95% CI: 36.72%-100%) as calculated using the PooledInfRate tool (Table 2). All egg pools from one parent tick tested positive (5 positive pools out of 5), whereas the egg pools from the other parent tick yield 4 positive and 1 negative pools (Table 3). After hatching, the positive rate for the larval pools was 32.63% (8 positive pools out of 16; 95% CI: 15.55%-63.54%), as determined using the PooledInfRate tool (Table 2). Notably, all the 8 positive larval pools originated from the same parent tick that produced the 5 positive egg pools, while all the 8 negative larval pools came from the other parent tick (Table 3).

## Discussion

Since its identification and isolation, *R. heilongjiangensis* has proven to be an important pathogen, widely distributed in Eastern Asia [8,15,17,18,24–27]. Our previous study identified a high prevalence of this pathogen in ticks attached to hedgehogs, likely attributable to cofeeding transmission [8]. To further understand the role of tick cofeeding in the transmission of SFGR, we established this model utilizing *R. heilongjiangensis*, ticks, and hedgehogs.

To create an infected tick population, we evaluated two mouse species: BALB/c and C3H/HeN. Both species have previously been used in SFGR infection models [19,21], but our current study revealed that C3H/HeN mice are more susceptible to *R. heilongjiangensis* infection, exhibiting more pronounced symptoms and higher bacterial loads in their organs. Additionally, bacteria DNA was observed in the infected C3H/HeN mice, making them suitable for establishing an infected tick population. However, low amount of *Rickettsia* was detected in the sera than in the tested organs, and the use of sera instead of whole blood or buffy coat for detection could be the reason. All engorged larvae that fed on the infected C3H/HeN mice tested positive for *R. heilongjiangensis*, confirming the successful establishment of an infected tick population.

After molting, the positive rate decreased from 100% in engorged larvae to 60% in molted nymphs. This decline suggests that 40% of the ticks did not successfully establish an infection. Further evaluation indicated that the infected tick population could effectively transmit *R. heilongjiangensis* to mice via biting, resulting in bacteremia, which suggests that the bacteria have established an infection in the ticks’ salivary glands. However, unlike the results seen with artificial abdominal infections in mice, *R. heilongjiangensis* was not detected in the livers and lungs of mice infected through tick bites.

Through cofeeding with infected nymphs, 75.61% of the uninfected nymphs that attached to mice and 100% of the uninfected adults that attached to hedgehogs became infected with *R. heilongjiangensis*. The high infection rate in the cofeeding adults aligns with the positive rates of ticks collected from wild hedgehogs, as noted in both our previous study (positive rate of 83.2%) and the current research (6/7 = 85.7%) [8]. These findings indicate that we have successfully simulated the infection process caused by tick cofeeding under laboratory conditions, supporting the hypothesis that the high positive rate of adult ticks attached to hedgehogs is likely a result of cofeeding.

Similar to the phenomenon observed in engorged larvae, the positive rate dramatically decreased (from 75.6% to 11.1%) when engorged nymphs molted to adults. This suggests that *Rickettsia* acquired through cofeeding does not consistently establish a firm infection in ticks, which may explain the low positive rates observed in free-living ticks.

In our laboratory simulations, we observed another interesting phenomenon: *R. heilongjiangensis* infection could lead to mortality in some adult ticks and cause eggs laid by infected adults to fail to hatch. The positive rate of the hatched larvae was notably lower than that of the eggs. Our data suggest that when 100 adult ticks are infected through cofeeding, approximately 40 ticks successfully produced normal eggs that can hatch into larvae, yielding a positive rate of 3.12%. Similar lethal effect of *Rickettsia rickettsii* on its tick vector *Dermacentor andersoni*, was also observed in a previous study [28]. Another study also confirmed that *R. rickettsii* infection impacted the fertility and fecundity of *Dermacentor variabilis*. The infection resulted in female ticks achieving smaller engorgement weights, being less efficient in converting their blood meal into eggs, and producing smaller egg clutches with a lower hatching rate [29]. In addition, the death of the engorged adult tick might result from the host’s immune reaction rather than the effect of *Rickettsia* infection. Although the mechanisms remain unclear, these factors could contribute to the low prevalence of positive ticks in the wild. A similar phenomenon of adult and egg mortality was observed in infected engorged adult ticks collected from wild hedgehogs. However, great individual variations were observed that all the larval pools from one infected adults were positive, and those from the other one were negative. The result of the latter adult tick is consistent with our laboratory simulation results that the positive rate through transovarial transmission dramatically decreased. The positive rate of the larval pools from the other adult tick was surprising. However, in the wild, given that ticks may carry a variety of microorganisms that affect their survival, development, and reproduction, it is normal to have some discrepancies with laboratory simulation results [29,30].

There are limitations to the present study. Firstly, the successful bite rates of ticks varied across different experiments, and the number of eggs laid by individual adults also fluctuated, potentially introducing bias into the experimental groups. Secondly, we used pooled samples for analyzing eggs and larvae, and while we employed a specific analysis tool for pooled samples, the calculated positive rates may deviate from the actual values. Thirdly, the positive rates produced by detecting engorged ticks could reflect the presence of infected blood meal rather than a true Rickettsial infection establishment in the ticks, and further study should be done to confirm the relationship between dynamic change of *Rickettsia* DNA copies and the infection establishment in ticks. Additionally, the mechanisms behind the observed phenomena are not fully understood; for instance, the reasons why some *R. heilongjiangensis*-infected adults died while others survived remain unclear.

In conclusion, this study established a model of SFGR transmission through tick-cofeeding on hedgehogs, simulating the natural transmission process. Ticks are reservoir host of *Rickettsia* and wild hedgehogs can get infection through *Rickettsia*-infected tick bite. Though the infection may be transient and not chronic, the hedgehogs play a role of “bridge” that horizontally transmits *Rickettsia* through cofeeding from infected ticks to uninfected ones. This is an amplification procedure. The high positive rate of *Rickettsia* in hedgehog-attached ticks results from this procedure, although infections acquired via this method do not maintain a consistently high positivity rate. Specifically, *Rickettsia* obtained from cofeeding or blood meals cannot successfully establish infections in all recipient ticks, and the positive rate declines significantly as they progress to subsequent developmental stages. Furthermore, adult ticks infected through cofeeding exhibit reduced infection rates in their offspring due to various factors, including tick mortality caused by rickettsiae, egg hatching failures, and low transovarial transmission rates. Moreover, under field conditions, infections from other pathogens may similarly lead to tick mortality and egg hatching failures. These results indicate the vertical transmissions, including both transstadial and transovarial transmissions, reduce the infection rate of ticks. Combining with the horizontal and vertical transmissions, the positive rates of *Rickettsia* maintain a dynamic equilibrium state. This study provides insights into the low prevalence of rickettsiae in nature and evaluates the role of cofeeding in the transmission of the pathogen among ticks. While tick-cofeeding on host animals is recognized as an important mechanism for amplifying SFGR prevalence, our findings suggest that its impact may not be as significant as previously thought.

## Supporting information

S1 Fig Detection of *R.* *heilongjiangensis* in the sera, skin (bite site), spleens, livers, lungs, and kidneys of mice on days 1, 6, and 12 post-attachment with infected nymphs, using semi-nested PCR targeting the 17-kDa gene. 1 to 8 on Days 1 and 6 represent heart, liver, spleen, lung, kidney, brain, skin, and serum, respectively; 1 to 8 on Day 12 represent heart, serum, skin, liver, spleen, lung, kidney, and brain, respectively; P, positive control; M, DNA marker. (PPTX)
