# Supplementary material for: Roles of tick-cofeeding hedgehogs in the natural transmission of spotted fever group Rickettsia
Source: PLoS Negl Trop Dis. 2025 Sep 15;19(9):e0013224. doi: 10.1371/journal.pntd.0013224 (PMC12435724; doi:10.1371/journal.pntd.0013224)
Supplement: S1 Fig — (PPTX) [file pntd.0013224.s001.pptx]

## Slide 1
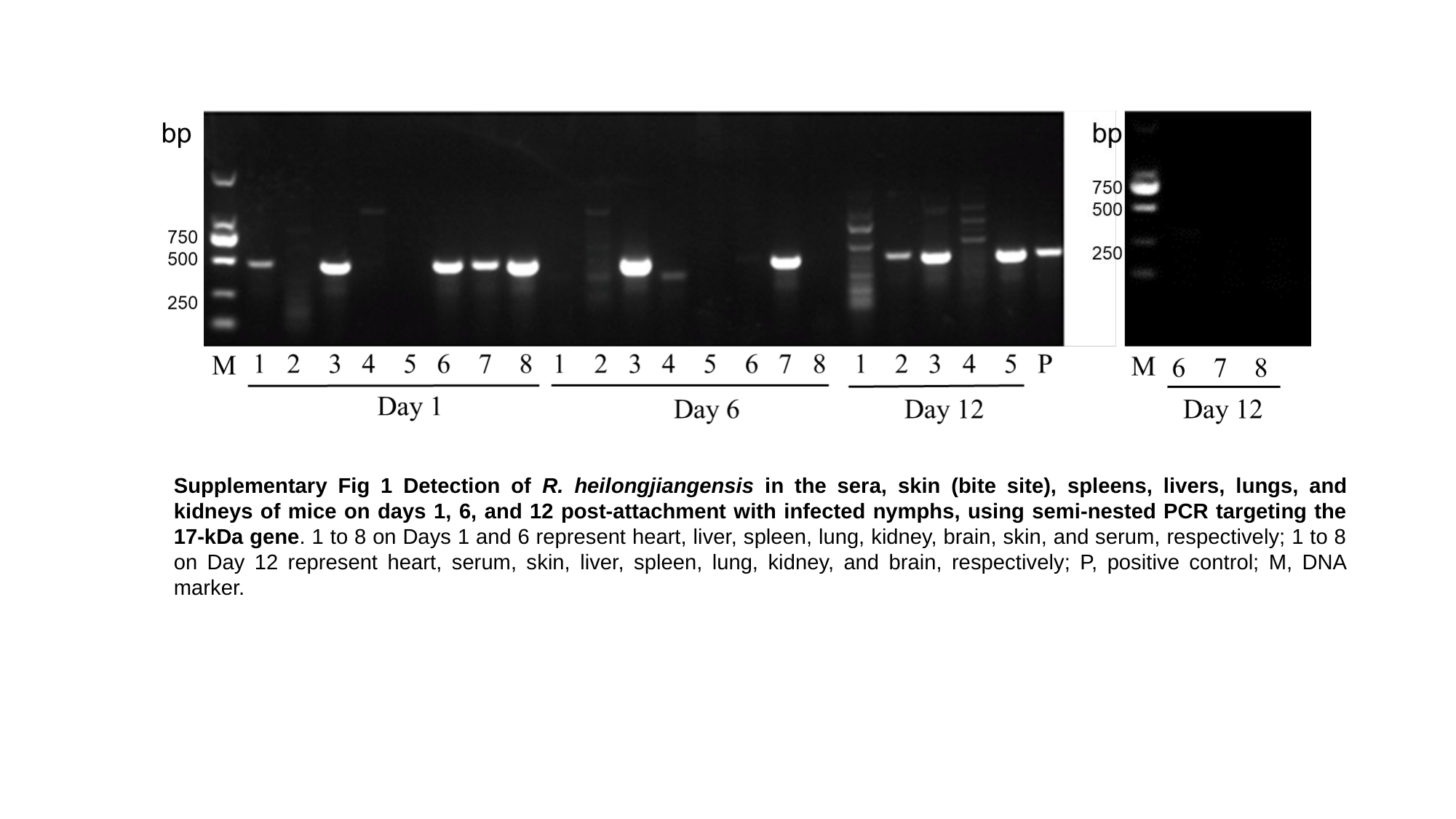

Supplementary Fig 1 Detection of R. heilongjiangensis in the sera, skin (bite site), spleens, livers, lungs, and kidneys of mice on days 1, 6, and 12 post-attachment with infected nymphs, using semi-nested PCR targeting the 17-kDa gene. 1 to 8 on Days 1 and 6 represent heart, liver, spleen, lung, kidney, brain, skin, and serum, respectively; 1 to 8 on Day 12 represent heart, serum, skin, liver, spleen, lung, kidney, and brain, respectively; P, positive control; M, DNA marker.
